# Nitrite stress increases staphylococcal enterotoxin C transcription and triggers the SigB regulon

**DOI:** 10.1093/femsle/fnac059

**Published:** 2022-07-26

**Authors:** Danai Etter, Ramona Büchel, Tabea Patt, Michael Biggel, Taurai Tasara, Nicole Cernela, Marc J A Stevens, Sophia Johler

**Affiliations:** Institute for Food Safety and Hygiene, University of Zurich, Winterthurerstrasse 272, 8057 Zurich, Switzerland; Institute for Food, Nutrition and Health, ETH Zurich, Schmelzbergstrasse 9, 8092 Zurich, Switzerland; Institute for Food, Nutrition and Health, ETH Zurich, Schmelzbergstrasse 9, 8092 Zurich, Switzerland; Institute for Food, Nutrition and Health, ETH Zurich, Schmelzbergstrasse 9, 8092 Zurich, Switzerland; Institute for Food Safety and Hygiene, University of Zurich, Winterthurerstrasse 272, 8057 Zurich, Switzerland; Institute for Food Safety and Hygiene, University of Zurich, Winterthurerstrasse 272, 8057 Zurich, Switzerland; Institute for Food Safety and Hygiene, University of Zurich, Winterthurerstrasse 272, 8057 Zurich, Switzerland; Institute for Food Safety and Hygiene, University of Zurich, Winterthurerstrasse 272, 8057 Zurich, Switzerland; Institute for Food Safety and Hygiene, University of Zurich, Winterthurerstrasse 272, 8057 Zurich, Switzerland

**Keywords:** stress response, superantigen, food intoxication, virulence gene regulation, *sec* variants, *Staphylococcus aureus*

## Abstract

Staphylococcal food poisoning is a common food intoxication caused by staphylococcal enterotoxins. While growth of *Staphylococcus aureus* is not inhibited by the meat-curing agent nitrite, we hypothesize that nitrite has an influence on enterotoxin C (SEC) expression. We investigated the influence of 150 mg/l nitrite on SEC expression at mRNA and protein level in seven strains expressing different SEC variants. Additionally, regulatory knockout mutants (*Δagr*, *ΔsarA*, and *ΔsigB*) of high SEC producing strain SAI48 were investigated at mRNA level. Our findings suggest that nitrite effectively increases *sec* mRNA transcription, but the effects on SEC protein expression are less pronounced. While *Δagr* mutants exhibited lower *sec* mRNA transcription levels than wildtype strains, this response was not stress specific. *ΔsigB* mutants displayed a nitrite stress-specific response. Whole genome sequencing of the strains revealed a defective *agr* element in one strain (SAI3). In this strain, *sec* transcription and SEC protein synthesis was not affected by the mutation. Consequently, additional regulatory networks must be at play in SEC expression. Comparison of our findings about SEC with previous experiments on SEB and SED suggest that each SE can respond differently, and that the same stressor can trigger opposing responses in strains that express multiple toxins.

## Introduction

Staphylococcal food poisoning (SFP) is amongst the most common food intoxications worldwide (Fetsch and Johler 2018). The EU reported 74 SFP outbreaks resulting in 1400 cases and 141 hospitalizations in 2019 (EFSA 2021), although the disease is likely heavily underreported (Hennekinne et al. 2012), and the USA estimate the yearly number of cases at 241 148 (Scallan et al. 2011). SFP is caused by growth of *Staphylococcus aureus* and its subsequent production of staphylococcal enterotoxins (SEs) in the food matrix. With 151 outbreaks reported in 2019 in the EU, meat and meat products were one of the main food vehicles implicated in food associated illness (EFSA 2021). Meat dishes have reportedly been responsible for SFP outbreaks (Johler et al. 2013, Mossong et al. 2015) and carcasses are commonly contaminated by *S. aureus* (Ebner et al. 2013, Morach et al. 2019). Although livestock does provide a potential entry point for *S. aureus* into the food chain, secondary contaminations by food handlers are more likely (Johler et al. 2011, Wattinger et al. 2012).

Usually, *S. aureus* is inhibited in growth by the surrounding microbiota. However, under stressful conditions such as acidic pH, inhibitory substances, or low a_w_, competitors are suppressed and *S. aureus* gains a competitive advantage and can outgrow them (Oberhofer and Frazier 1961). Once SEs are produced in the food matrix, heat treatment will not render them inactive as they are highly heat stable. Thus, food with inactivated *S. aureus* can still lead to intoxication (Le Loir et al. 2003). So far, the classical SEs SEA–SEE, as well as the newly described SEs SEG–SE*l*Z have been characterized. staphylococcal enterotoxin C (SEC) stands out as particularly diverse with the human variants SEC_1–4_ and the animal variants SEC_bovine_ and SEC_ovine_, which can all be implicated in SFP (Etter et al. 2020). In addition, SEC is expressed in up to 10 times higher amounts than other SEs (Spaulding et al. 2013). Therefore, SEC is of particular interest regarding SE expression under conditions relevant to food production.

One strategy to minimize microbial risks associated with food such as intoxications are hurdle technologies (Leistner 2000). In this approach, different preservation methods that by themselves do not suffice for pathogen inhibition such as salting, drying, or heating are combined to improve overall food safety. A commonly added preservative for meat and meat products is nitrite (NaNO_2_). It contributes to sensory properties, color stability, and food safety in cured meats. The EU allows a maximum of 150 mg/kg nitrite in meat products with the exception of several traditional cured meat products that may contain up to 175 mg/kg (EUR-Lex 2011, Mortensen et al. 2017). However, consumers nowadays increasingly demand more natural, fresh, and minimally processed foods without additives. The use of nitrite in meat as a curing agent has raised particular public concern because nitrite was shown to be a precursor of nitrosamines, which are known to be carcinogenic (Pegg and Shahidi 2004). Therefore, lowering the maximum amount of nitrite allowed in foods is currently being discussed (Mortensen et al. 2017). Limiting the use of nitrite might, however, enhance pathogen growth or lead to increased toxin concentrations. SE production was already demonstrated in meat matrices containing nitrite (Wallin-Carlquist et al. 2010b, Márta et al. 2011, Susilo et al. 2017).

Nitrite acts as a precursor of nitric oxide (NO·), which in turn generates peroxynitrite (ONOO-). This reactive molecule is capable of oxidizing and nitrating DNA, proteins, and lipids through direct or indirect mechanisms (Majou and Christieans 2018). Bacteria react to this stress by activating oxidative/pH stress responses and changes in their respiratory activity. However, the exact mechanisms of nitrite stress and its potential influence on SE production remain unclear.

Like many SEs, SEC is regulated by the accessory gene regulator (*agr*; Regassa et al. 1991). Additional regulatory genes such as sigma factor B (*sigB*) and the staphylococcal accessory regulator (*sarA*) are likely involved under stress conditions (Schmidt et al. 2004, Fisher et al. 2018). The Agr system causes a strategic switch from an early establishment phase to a late attack phase by activating a plethora of virulence genes. SarA is a positive regulator and SigB a negative regulator of the Agr system. Studies on SEB and SED that fall under the same regulon as SEC revealed that mild nitrite stress (150 mg/l) has negligible impact on *S. aureus* growth (Sihto et al. 2016, 2017) and SEB and SED protein levels were not affected by nitrite concentrations below 200 mg/l (McLean et al. 1968, Sihto et al. 2016, Schelin et al. 2017). So far, the influence of nitrite on SEC expression has not been investigated.

We assessed the influence of 150 mg/l nitrite stress on *sec* expression in seven strains from different origins and with different SEC variants and *sec* gene promoters (promoter variants v1–v4; Table 1). Quantification at both mRNA and protein level enabled us to determine whether gene regulation as a nitrite stress response is transcriptional or post-transcriptional. We used 150 mg/l nitrite containing medium to represent concentrations encountered in cured meat products. The concentration of nitrite was chosen to provide the same amount of total nitrite as would be available in 1 kg of cured meat. Our findings contribute to a better understanding of matrix–pathogen interaction, i.e. needed to adapt food production parameters and protect consumer health.

**Table 1.** Overview of *S. aureus* strains used in this study including their SEC variants, origin, and assignment to clonal complexes.

Strain	Protein variant	*sec* promoter variant	Origin	Clonal complex, spa type	Reference
BW10	SEC_2_	sec_p_ v1	SFP	CC45, t383	Johler et al. (2011)^1^
NB6	SEC_2_	sec_p_ v1	SFP	CC45, t6969	Johler et al. (2011)^2^
SAI3	SEC_1_	sec_p_ v3 (H-EMRSA-15)	Human infection	CC8, t148	Wattinger et al. (2012)
SAI48	SEC_2_	sec_p_ v1 (79_S10)	Human infection	CC5, t002	Wattinger et al. (2012)
SAR1	SEC_bovine_	sec_p_ v2	Bovine mastitis milk	CC151, t529	Johler et al. (2011)
SAR38	SEC_bovine_	sec_p_ v2	Bovine mastitis milk	CC151, t529	Johler et al. (2011)
OV20	SEC_ovine_	sec_p_ v4	Ovine	CC133, t4735	Guinane et al. (2010)
SAI48Δagr::erm	SEC_2_	sec_p_ v1 (79_S10)	Human infection	CC5, t002	Sihto et al. (2017)
SAI48ΔsarA::tet	SEC_2_	sec_p_ v1 (79_S10)	Human infection	CC5, t002	Sihto et al. (2017)
SAI48ΔsigB::tet	SEC_2_	sec_p_ v1 (79_S10)	Human infection	CC5, t002	Sihto et al. (2017)

1 BW10 = SFP18.

2 NB6 = SFP12.

## Materials and methods

### Bacterial strains, growth conditions, and sample collection for sec mRNA and SEC protein quantification

All *S. aureus* strains and their respective SEC variants in this study are listed in Table 1. The strains were grown in LB medium (BD, Pont-de-Claix, France) (nonstress control conditions) and in LB supplemented with 150 mg/l sodium nitrite (Sigma Aldrich, Buchs, Switzerland; 0.77 M). Nitrite stress conditions encountered in food were mimicked by adjusting to a concentration found in cured meat. All media were sterile filtered and stored at 4°C.

Strains BW10, NB6, SAI3, SAI48, SAR1, SAR38, and OV20 were grown and sampled according to procedures previously described in Etter et al. (2021). Briefly, colonies from 5% sheep blood agar were cultured in LB broth (16 h at 37°C and 125 rpm). After washing the cultures in 0.85% NaCl suspension (centrifugation and resuspension in 0.85% NaCl solution), 50 ml of medium (LB and LB + nitrite) were inoculated with varying amounts of washed culture to achieve a final OD_600_ of 0.05. Cultures were incubated at 37°C at 125 rpm and sampled after 4, 10, and 24 h. A total of three independent biological replicates were collected. RNAprotect®Tissue Reagent (Qiagen, Hilden, Germany) was used for mRNA sample stabilization. Low protein binding microcentrifuge tubes (Thermo Scientific, Waltham, MA, USA) were used for protein sample collection.

Growth curves (Figure S1, Supporting Information) were evaluated by measuring OD_600_ in 200 μl in 96-well plates in a Synergy plate reader (Biotek, Winooski, VT, USA) at 37°C. Wells were inoculated with varying amounts of washed culture to obtain a starting OD_600_ of 0.05. Nitrite stress conditions (LB + 150 mg/l nitrite) were compared to control conditions (LB medium).

### Regulatory knockout mutants

Strains SAI48Δagr::erm, SAI48ΔsarA::tet, and SAI48ΔsigB::tet (Table 1) were generated and qPCR experiments performed according to Sihto et al. (2017). Phage 80α was used to transduce the deleted regulatory elements from RN4220 into SAI48 as previously described (Charpentier et al. 2004, Sihto et al. 2015, 2017). Briefly, phage prep was generated according to Bose (2014) by adding 10 μl of 10^10^ phage to a fresh culture of the respective RN4220 knockout strain and incubating it for 5–6 h or until lysis. The phage prep was then titered. Subsequently, a fresh culture of recipient strains was inoculated with phage prep at an MOI of 0.1 for 45 min at 30°C. The transformed strains were then plated on selective agar containing the appropriate antibiotics.

For qPCR experiments, LB and LB supplemented with nitrite were inoculated with 10^−3^ dilutions of washed overnight cultures (50 μl) and incubated at 37°C, 225 rpm. RNA extraction and qPCR experiments were performed according to sections “RNA extraction” and “Reverse transcription and quantitative real-time PCR.” For comparison of the *sec* mRNA expression in the isogenic mutants with the wild type (wt) SAI48 under both control conditions and nitrite stress, the following formula was used:

$$\begin{equation*}
{2}^{ - \left( {\left( {c{t}_{ref\left( {SAI48} \right)} - c{t}_{target\left( {SAI48} \right)}} \right) - \left( {c{t}_{ref\left( {isogenicmutant} \right)} - c{t}_{target\left( {isogenicmutant} \right)}} \right)} \right)},
\end{equation*}$$

where ref (SAI48) is the reference gene *rho* or *rplD* in SAI48 and target (SAI48) is the target gene *sec* in SAI48. Ref (isogenic mutant) stands for the reference gene *rho* and *rplD* of the isogenic mutants and target (isogenic mutant) is the target gene *sec* in the isogenic mutant. Statistical analysis was done using R (R-project.org) in RStudio (Version 1.4.1106, © 2009–2021 RStudio, PBC). To ensure normal distribution the fold change was log10-transformed. A linear mixed-effect model with three-way interaction was fitted using lmer. *Post hoc* analysis was performed using lsmeans (least-squares means). Results were considered significant if *P* < .05.

### RNA extraction

RNA extraction was performed with the RNeasy mini Kit Plus (Qiagen) as previously described (Sihto et al. 2014, Etter et al. 2021) and quantified with Quantifluor (Promega, Madison, WI, USA). Quality control was performed by the Agilent 2100 Bioanalyzer (Agilent Technologies, Waldbronn, Germany). Samples were included in the study if they met the inclusion criteria of RNA integrity numbers > 6. RNA integrity numbers ranged from 6.3 to 8.2. Further details are provided in Table S1 (Supporting Information).

### Reverse transcription and quantitative real-time PCR

All RNA samples were subjected to reverse transcription and qRT-PCR as previously described (Etter et al. 2021). Relative expression of the target gene *sec* was normalized using the housekeeping genes *rho* and *rplD* (Sihto et al. 2014). Ct values were determined using the Lightcycler®Software version 1.1.0.1320 (Roche, Basel, Switzerland). Data was expressed as Δct values (target-reference). mRNA data was log transformed and analyzed via two-way ANOVA and *post hoc* Tukey’s multiple comparisons. Results were regarded as significant if *P* < .05.

### Protein quantification

An Enzyme-linked immunosorbent assay (ELISA) was performed as previously described (Etter et al. 2021). The protocol was based on Poli et al. (2002) with some modifications according to Wallin-Carlquist et al. (2010a). Immulon® 2HB “U” Bottom Microtiter® Plates (96 wells, Thermo Scientific) were coated with 100 μl/well of 2 μg/ml of sheep Anti-SEC IgG (Toxin Technology, Inc., Sarasota, FL, USA) in coating buffer (0.1 M Na2CO3, pH 9.6) and incubated at 37°C overnight. Wells were incubated with 185 μl PierceTM Protein-Free (PBS) blocking buffer (Thermo Scientific) for 1 h at 37°C and for 1 h at 4°C. The wells were washed twice with 185 μl wash buffer (10 mM phosphate-buffered saline (PBS), 0.05% Tween 20 (Sigma-Aldrich, Switzerland)) and incubated with 185 μl wash buffer in a shaking plate (5 min, 300 rpm) twice. The standard curves were prepared by diluting SEC_2_ (Toxin Technology, Inc.) toxin stock solution (100 μg/ml) in a range of 0.0175–1.25 ng. Standards and samples were transferred to the wells (100 μl/well) and incubated at 37°C for 1.5 h. The plates were washed as described above. The detection sheep biotinylated Anti SEC IgG (Toxin Technology, Inc.) was diluted 1:2000 in assay buffer [50 mM PBS, 0.01% bovine serum albumin (BSA; Sigma-Aldrich, Germany), 0.1% Tween 20 (Sigma-Aldrich), and 0.01% thimerosal (Sigma-Aldrich) including 1% milk powder (Difco, Switzerland)] and 100 μl of the solution was added to each well and incubated at 37°C for 1 h. The plate was washed again as described before. Neutravidine-linked alkaline phosphatase (Thermo Scientific) was diluted 1:1000 in assay buffer containing 1% milk powder. A volume of 100 μl/well of this solution was incubated at 37°C for 30 min. A volume of 100 μl/well of substrate (SIGMA FastTM p-Nitrophenyl phosphate tablet set, Sigma-Aldrich) was added after a final wash and incubated for 45 min at room temperature in the dark. Absorbance at 405 nm was measured exactly after 45 min in a plate reader (Synergy, Biotek). ELISA measurements were performed in duplicates. Statistical analysis was performed with RStudio 1.3.1093 and GraphPad Prism 9.2.0. protein data was log transformed and analyzed via two-way ANOVA and *post hoc* Tukey’s multiple comparisons. Results were regarded as significant if *P* < .05.

### DNA extraction and WGS

Genomic DNA was extracted using the DNeasy Blood and Tissue Kit (Qiagen). Libraries were prepared with the Nextera DNA Flex Library Preparation Kit (Illumina) and sequencing performed on the Illumina MiniSeq platform with 2 × 150 bp paired-end chemistries. and quality metrics computed using FastQC v0.11.9 (bioinformatics.babraham.ac.uk/projects/fastqc/). Draft genomes were assembled using SPAdes v3.14.1 (Bankevich et al. 2012) implemented in shovill v1.1.0 (github.com/tseemann/shovill) and annotated with prokka v1.14.6 (Seemann 2014).

## Results and discussion

### sec mRNA transcription increased under nitrite stress in some strains

**Figure 1.**
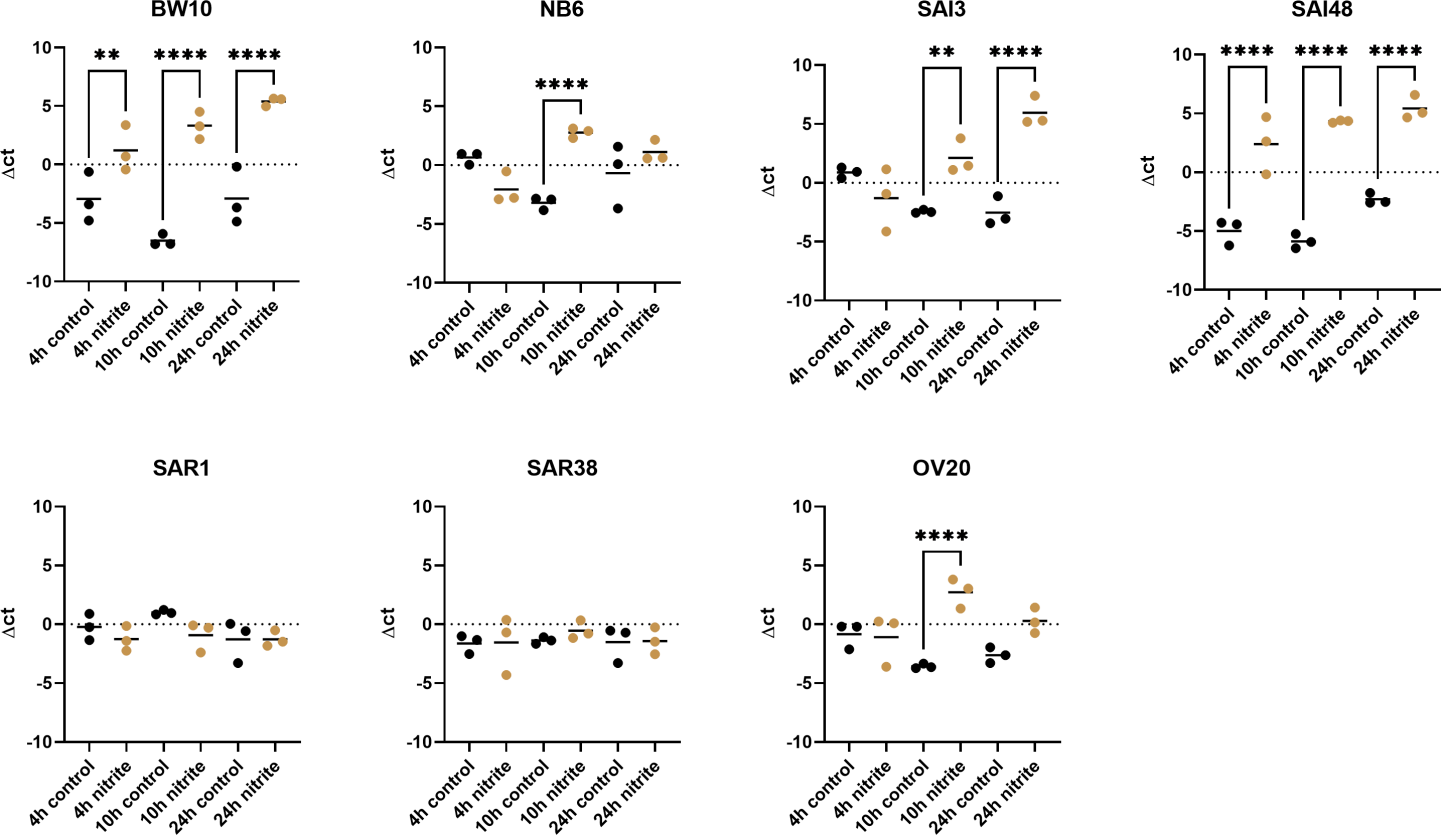
Effect of nitrite stress on sec mRNA levels in seven *S. aureus* strains (BW10, NB6, SAI3, SAI48, SAR1, SAR38, and OV20). mRNA Levels were measured by qRT-PCR. qPCR Δct values in exponential (4 h), early stationary (10 h), and late stationary phase (24 h) in LB and LB + 150 mg/l nitrite for each time point and strain. Control conditions in black, nitrite stress conditions in orange. Target mRNA (sec) was normalized to two reference genes rho and rplD. Statistically significant changes in sec mRNA levels in LB + 150 mg/l nitrite compared to LB (*P* < .05) are marked by asterisks (* = *P* < .05, ** = *P* < .01, *** = *P* < .001, and ^****^ = *P* < .0001).

Different *S. aureus* strains were subjected to nitrite stress and *sec* mRNA levels were measured in exponential (4 h), early stationary (10 h), and late stationary phase (24 h) across seven *S. aureus* strains and expressed normalized to growth of the respective strain. No differences in growth rate between nitrite stress and control conditions could be observed (Figure S1, Supporting Information). The expression of *sec* mRNA under nitrite stress was higher compared to control conditions in all growth phases for strains BW10 (CC45) and SAI48 (CC5; Fig. 1). Strains NB6 (CC45), OV20 (CC133), and SAI3 (CC8) had increased expression levels only in early stationary (10 h) and/or late stationary (24 h) phase. A total of five out of seven strains (BW10, NB6, SAI3, SAI48, and OV20) exhibited significantly altered expression. Isolates BW10, NB6, SAI48 (all SEC2, v1), SAI3 (SEC_1_, v3), and OV20 (SEC_ovine_, v4) showed increased levels of *sec* transcripts in at least one growth phase. The two bovine strains (SAR1, SAR38, both CC151, both SEC_bovine_, and v2) were not significantly affected by nitrite stress. Contrary to an increase in *sec* transcription under nitrite stress previous experiments under lactic acid stress and NaCl stress had revealed downregulation of *sec* at mRNA level for both stress conditions at several timepoints (Etter et al. 2021, 2022).

Other SEs that fall under the same regulon (*agr*) as SEC have been investigated under nitrite stress. Similar to the increase in *sec* mRNA transcription we observed, previous experiments demonstrated an induction of *sed* mRNA transcription under 150 mg/l nitrite stress, especially in later growth phases (Sihto et al. 2016). Strain SAI48 that was included in both studies and displayed similar expression patterns for *sec* and *sed*. Another study revealed decreased *seb* promoter activity under 150 mg/l nitrite stress, mainly in exponential phase (6 h/10 h; Sihto et al. 2017). This suggests that each SE can respond differently, and that the same stressor can trigger opposing responses in strains that express multiple toxins.

### SEC protein expression does not reflect mRNA expression patterns

**Figure 2.**
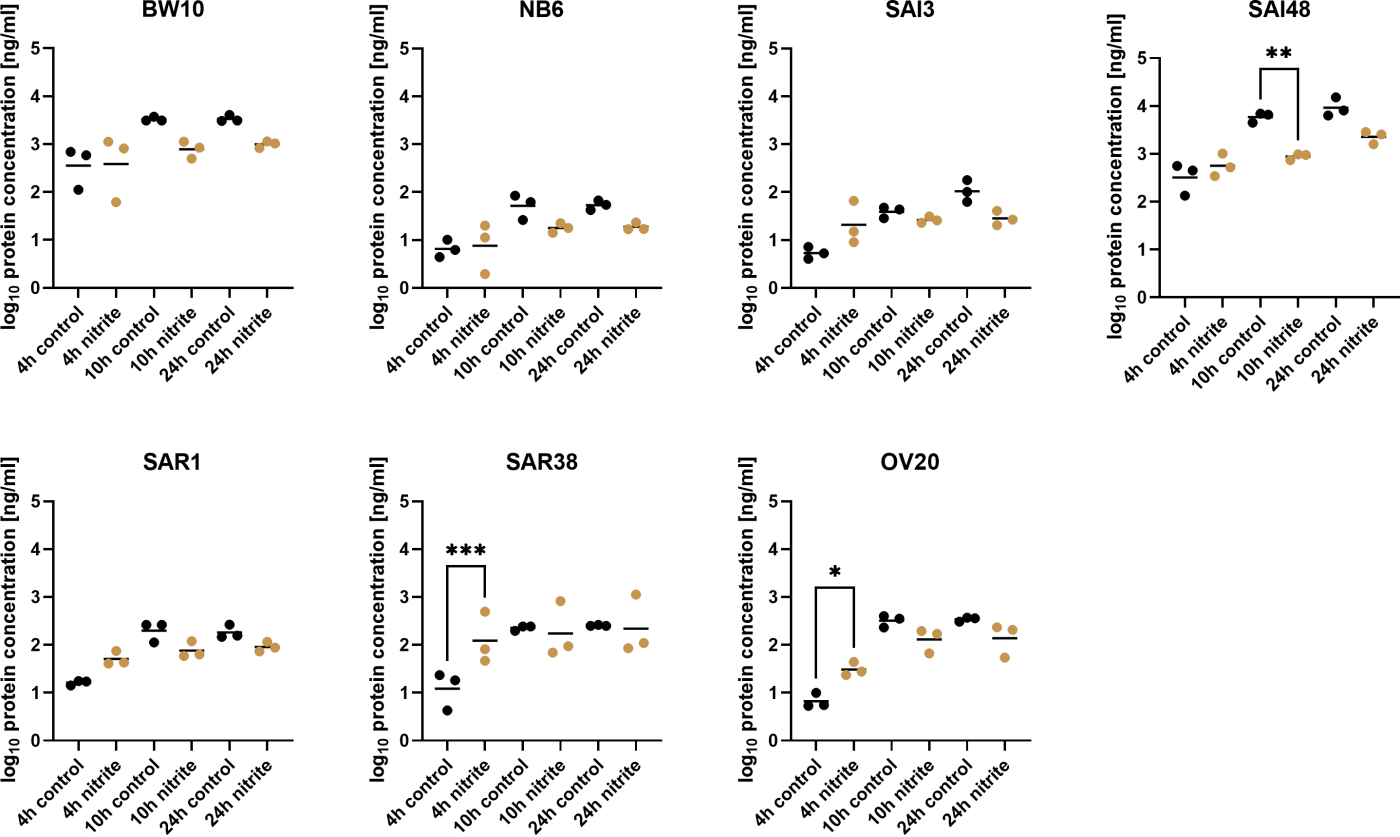
Effect of nitrite stress on SEC protein levels in seven *S. aureus* strains (BW10, NB6, SAI3, SAI48, SAR1, SAR38, and OV20). Levels were measured by ELISA. log10 values of protein concentration in ng/ml in exponential (4 h), early stationary (10 h), and late stationary phase (24 h) in LB and LB + 150 mg/l nitrite for each time point and strain. Control conditions in black, nitrite stress conditions in orange. Target mRNA (sec) was normalized to two reference genes rho and rplD. Statistically significant changes in sec mRNA levels in LB + 150 mg/l nitrite compared to LB (*P* < .05) are marked by asterisks (* = *P* < .05, ** = *P* < .01, *** = *P* < .001, and ^****^ = *P* < .0001).

In addition to *sec* mRNA transcript levels, SEC protein concentrations were measured by ELISA in exponential (4 h), early stationary (10 h), and late stationary phase (24 h). Expression patterns did not fully reflect the pronounced increase in mRNA expression observed in strains BW10, NB6, SAI3, SAI48, and OV20 (Fig. 2). On the contrary, SAI48 even had reduced SEC levels in exponential phase, although this strain exhibited significantly increased *sec* mRNA levels in all growth phases (Fig. 1). All strains showed increased SEC protein levels in early exponential phase (4 h), but results were only significant for SAR38 and OV20 (Table 2). Overall, strains SAI3, SAR1, SAR38, and OV20 displayed an increase in SEC under nitrite stress, mostly because of a pronounced increase after 4 h (Table 2), while BW10, NB6, and SAI48 had reduced levels of SEC. Strain SAI48 and two other strains had previously been shown to express significantly less SED under nitrite stress (Sihto et al. 2016), which was only partly reflected in the present study on SEC. Hence, the type of SE, i.e. being investigated seems to influence the effect of an applied stress or on enterotoxin production. Our study suggests that nitrite does significantly increase *sec* mRNA transcription in many strains, but this effect does not fully carry over to the produced amounts of protein. Post-transcriptional modifications could, therefore, be involved in SEC expression under nitrite stress. Based on these findings, addition of nitrite to food might not contribute to lower enterotoxin levels and might potentially even pose a threat to consumers by elevating SEC levels in some *S. aureus* strains especially in early growth phases. Additional hurdles such as pH or drying may be necessary to assure food safety (Wallin-Carlquist et al. 2010b, Bang et al. 2008).

**Table 2.** Effect of nitrite on SEC protein expression as measured by an ELISA assay. Values are given in ng/ml and the effect of nitrite is calculated in % relative to the control condition.

Strains	SEC produced under nitrite stress (ng/ml)						Effect of nitrite [%]			
	4 h control	10 h control	24 h control	4 h NaNO_2_	10 h NaNO_2_	24 h NaNO_2_	4 h	10 h	24 h	Sum
BW10	462 ± 307	3325 ± 360	3410 ± 562	670 ± 449	826 ± 258	999 ± 129	45	−75	−71	−101
NB6	7 ± 3	58 ± 29	54 ± 12	11 ± 7	18 ± 3	19 ± 3	61	−68	−65	−73
SAI3	6 ± 2	40 ± 10	114 ± 59	30 ± 26	27 ± 3	29 ± 8	447	−33	−74	339
SAI48	380 ± 220	5988 ± 1317	9868 ± 4688	626 ± 284	886 ± 106	2332 ± 539	65	−85	−76	−97
SAR1	16 ± 2	213 ± 87	189 ± 65	53 ± 16	81 ± 28	92 ± 18	224	−62	−51	111
SAR38	15 ± 10	228 ± 27	254 ± 7	207 ± 202	328 ± 349	439 ± 484	1248	44	73	1365
OV20	7 ± 3	328 ± 87	345 ± 34	32 ± 9	144 ± 56	165 ± 79	357	−56	−52	249

### SigB regulates sec expression under nitrite stress

**Figure 3.**
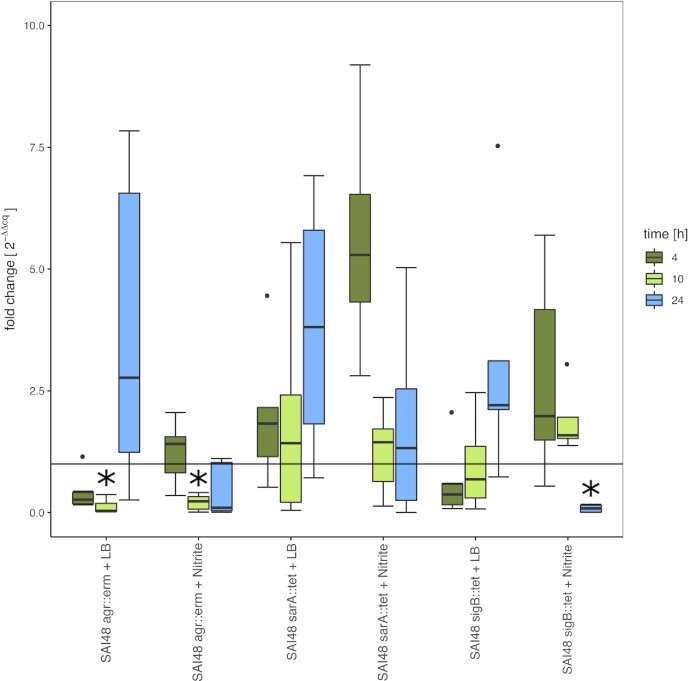
Effect of nitrite stress on sec mRNA levels in SAI48Δagr::erm, SAI48ΔsarA::tet, and SAI48ΔsigB::tet compared to the wt strain SAI48wt. Levels were measured by qRT-PCR expressed as fold change 2^–ΔΔct^. qPCR Δct values in exponential (4 h), early stationary (10 h), and late stationary phase (24 h) in LB and LB + 150 mg/l nitrite for each time point and strain always in relation to the respective levels in SAI48wt under either control or nitrite stress conditions. Target mRNA (sec) was normalized to two reference genes rho and rplD. Outliers are designated by single black dots. Statistically significant changes in sec mRNA levels in knockout strains compared to SAI48wt (*P* < .05) are marked by asterisks. The horizontal line indicates a fold change of 1 corresponding to the same level of expression as in SAI48wt.

The highest SEC toxin producing strain SAI48 (Table 2) was used to investigate the influence of regulatory genes on *sec* mRNA transcription under nitrite stress. The expression of *sec* in Δ*agr*, Δ*sarA*, and Δ*sigB* knockout mutants was compared to that of SAI48wt strain at all time-points (4, 10, and 24 h; Fig. 3). Loss of *agr* led to significantly lower *sec* transcription compared to wt levels at 10 h under control and nitrite stress conditions. The downregulation was similar in both conditions and hence not stress specific. Loss of *sarA* did not significantly influence *sec* transcription under either control or nitrite stress. A lack of *sigB* on the other hand generated divergent responses under the two different conditions. Under control conditions, lack of *sigB* led to lower *sec* transcription in early growth phases (4 and 10 h) and elevated ones at 24 h. In contrast, under nitrite stress, *sec* transcription was increased at 4 and 10 h, but significantly decreased after 24 h compared to wt. *Agr* does have an influence on *sec* transcription, but its effect does not depend on the applied stress condition. Conversely, s*igB* seems to react specifically to nitrite stress and might outweigh the influence of *agr*.

In a previous study, SAI48 regulatory knockouts were investigated under nitrite stress in terms of SED protein expression. There, loss of *sigB* had led to an increase of SED under control conditions, but not under nitrite stress (Sihto et al. 2016). However, SED expression was only investigated at protein level and results can, therefore, not be compared directly.

**Figure 4.**
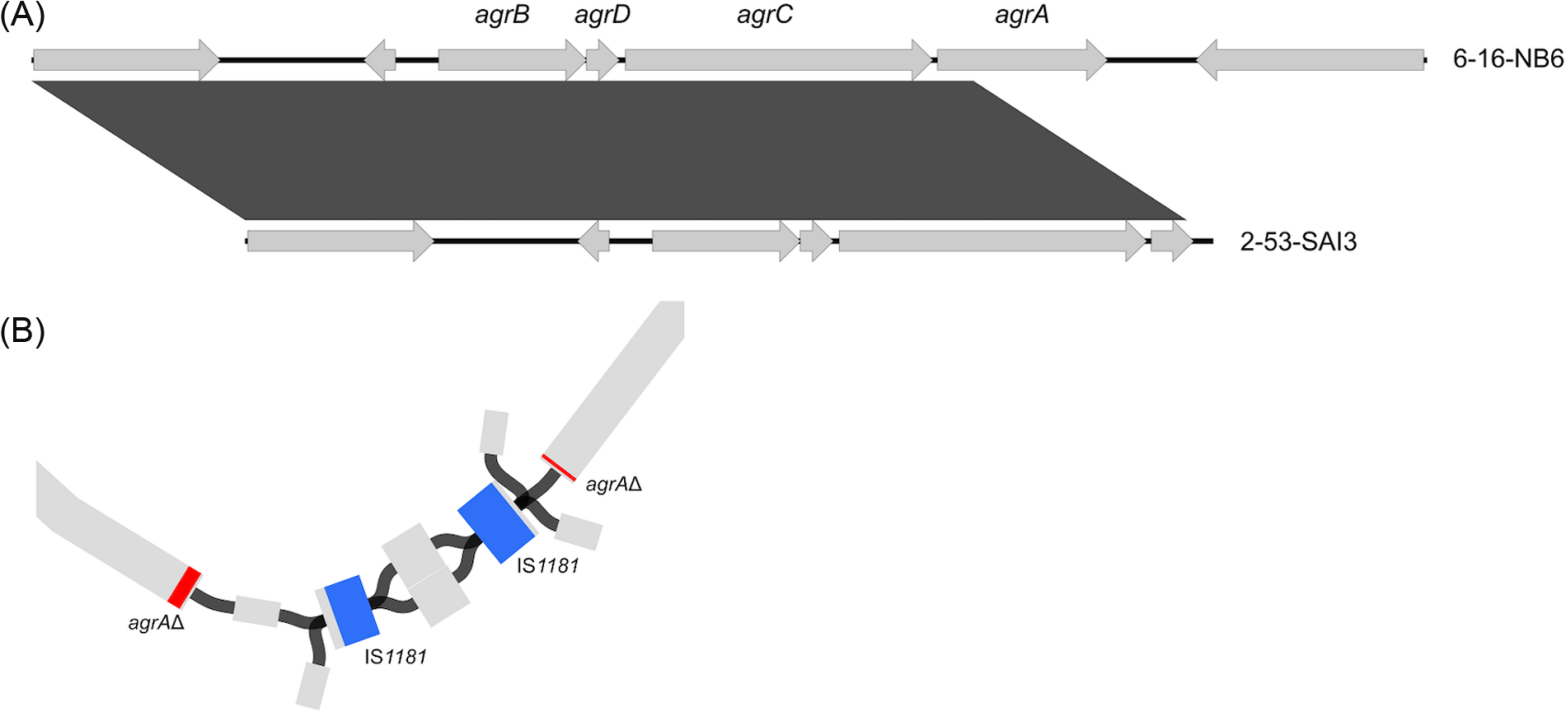
Organization of the accessory gene regulator (agr) locus and disruption of agrA in isolate SAI3. (A) Comparison of the agr locus comprising agrB, agrD, agrC, and agrA in isolates NB6 and SAI3. Shaded boxes between sequences indicate homologous regions (> 90% sequence identity). In SAI3, partial agrA fragments were identified on distinct assembly contigs. The figure was generated with Easyfig 2.1 (Sullivan et al. 2011) and Inkscape v1.0.1. (B) Visualization of the SPAdes assembly graph around agrA fragments of isolate SAI3. Contigs (in gray) containing agrA fragments (red) are interrupted by contigs harboring an IS1181 insertion sequence (blue, combined length 1.7 kb). The figure was generated in Bandage v0.8.1 (Wick et al. 2015).

Whole genome sequencing (WGS) analysis revealed a truncated *agrA* gene in strain SAI3. Assembly graph visualization suggested that the gene was disrupted by the insertion of a transposable IS*1181* element (Fig. 4). Consequently, RNAIII driven inactivation of the repressor of toxins (Rot) is impossible, unless RNAIII can be activated via alternative pathways (Bronesky et al. 2016). Hence, significantly less SEC production would be expected in this strain. However, our results showed that SAI3 produced equal amounts of SEC as NB6, which possesses an intact *agr* locus. In addition, transcriptional regulation under nitrite stress resembled that of NB6 and OV20. Both under optimal conditions and under nitrite stress, SAI3 seems able to still produce adequate amounts of SEC although *agrA* is not functional. At least for some strains SEC expression is likely regulated via *agr*-independent pathways.

## Conclusion and outlook

Nitrite is a widely used food preservative in meat products to prevent bacterial growth. In this study, we investigated the effect of sodium nitrite on *sec* mRNA and SEC protein levels in seven *S. aureus* strains. Nitrite stress led to either increased or unchanged *sec* mRNA transcription in all strains. SEC protein levels were not affected in most strains and led to increased SE concentration in some strains. This points towards a likely post-transcriptional regulation of SEC under nitrite stress. The comparison of our findings about SEC with previous experiments about *seb* and *sed* expression suggests that each SE can respond differently, and that the same stressor can trigger opposing responses in strains that express multiple toxins. Additionally, regulatory knockout mutants (*Δagr*, *ΔsarA*, and*ΔsigB*) of high SEC producing strain SAI48 were investigated at mRNA level under nitrite stress and control conditions. *Δagr* mutants had lower *sec* mRNA transcription than wt strains under nitrite and control conditions. *ΔsigB* mutants displayed opposed behavior under stress conditions compared to control conditions. *agr* does have an influence on *sec* transcription, but its effect does not depend on the applied stress condition. Conversely, s*igB* seems to react specifically to nitrite stress and might outweigh the influence of *agr*. WGS analysis of the wt strains revealed a defective *agr* element in the human isolate SAI3. The defective *agr* element in SAI3 did not influence *sec* transcription or SEC protein expression. Consequently, additional regulatory networks must be at play in SEC expression. However, to draw final conclusions regarding regulatory networks more knockout experiments are needed. Based on these findings, addition of nitrite to food, therefore, does not contribute to lower SEC levels and could even pose a potential threat to consumers by elevating SEC levels. Ultimately, our results demand verification in a suitable food model.

## Data availability

Genome assemblies generated as part of this study are available under BioProject number PRJNA789445. Genome accession numbers of all investigated isolates are listed in Table S2 (Supporting Information).

## Authors’ contributions

S.J., T.T., and D.E. contributed to the conception and design of the study. D.E., R.B., and T.P. analyzed the data. D.E. wrote the first draft of the manuscript. D.E., R.B., N.C., M.B., M.S., and T.P. were responsible for data acquisition. D.E., S.J., and T.T. wrote sections of the manuscript. All authors contributed to the manuscript revision, read, and approved the submitted version.

## Supplementary Material

fnac059_Supplemental_File
